# The mediating role of psychological commitment between recreation specialization and life satisfaction: Evidence from Xiamen Marathon runners

**DOI:** 10.3389/fpsyg.2022.1006289

**Published:** 2022-11-08

**Authors:** Haibo Tian, Wenting Zhou, Yajun Qiu

**Affiliations:** ^1^Department of Physical Education, School of Teacher Education, Shaoxing University, Shaoxing, Zhejiang, China; ^2^Department of Physical Education, College of Education, Zhejiang University, Hangzhou, Zhejiang, China

**Keywords:** recreation specialization, life satisfaction, psychological commitment, social support, marathon tourists

## Abstract

Although previous research spared no efforts to explain the life satisfaction of marathon runners, little was known about the relationship between recreation specialization (RS), life satisfaction (LS), psychological commitment (PC), and social support (SS). This study examines (i) how the dimension of RS (i.e., behavior, cognition, and affect) influences runners’ PC and LS, and (ii) the mediating effects of PC and the moderating effects of SS on the relationship between behavior, cognition, affect, and LS. The results showed that behavior (*β* = 0.15, *p* < 0.01), cognition (*β* = 0.35, *p* < 0.001), affect (*β* = 0.28, *p* < 0.001), and PC (*β* = 0.59, *p* < 0.001) had significant and positive impacts on runners’ LS; PC (Indirect path coefficient = 0.09 ~ 0.20) mediated the relationship between the dimensions of RS and LS. In addition, the results also confirmed the moderating effects of SS (*β* = 0.10, *p* < 0.05) between affect dimension and LS. These findings offered more evidence for understanding how RS dimensions and PC influence runners’ LS. Future research should integrate runner’s leisure experiences to better understand the results revealed in this study.

## Introduction

“*I run marathon as an excuse to travel to new places, meet new people and try out new food. Maybe I do not need an excuse to do all these things, but I think I may be hooked on running marathon now!*” (Mary, 2013).

With the rapid growth of marathon event in China, marathon event tourism is being studied in great demand in the leisure field (Wang et al., 2018). Statistical data showed that approximately 1,828 marathon events were held in 337 cities across China in 2019, attracting more than 7.13 million participants which represented an increase of 1.29 million participants compared with the number in 2018 (Chinese Athletica Association, 2020). Individuals were keen to travel away from the place of residence, and regarded harvesting marathon event as a key leisure pursuit (Sato et al., 2016). Previous studies found that marathon event brand and destination image were the significant predictors of runners’ willingness to revisit (Hallmann and Breuer, 2010; Huang et al., 2015). Moreover, other factors such as destination attachment, event satisfaction, and travel distance can also influence individual’s behavioral intention (Hallmann and Wicker, 2012; Filo et al., 2013).

Bryan (1977, 2000) developed a conceptual framework named “recreation specialization (RS)” to reflect various behaviors that people had showed during participating in leisure sport activities. Along with the progress of runner’s leisure career, they usually exhibited a series of obvious characteristics related to RS. As mentioned in the study of Green and Jones (2005), sport tourism can provide serious participants with (i) a way to construct their leisure identity, (ii) a time and place to express them sharing the skill and knowledge of the activity, and (iii) a way to record their leisure career. Existing literature (Heo and Lee, 2010; Kim et al., 2011; Yang et al., 2019) also provided some evidence to support the role of RS on life satisfaction (LS). For example, Yang et al. (2019) confirmed that the core devotees of sport club reported experiencing higher degree of happiness, life satisfaction, and health when compared to those moderate ones. In a longitudinal study, scholars indicated that distance running events participation exerted a positive influence on participants’ evaluations toward their lives (Sato et al., 2015).

While the previous study indicated that RS had a direct effect on individual’s leisure satisfaction (Matsumoto et al., 2018; Kwon et al., 2021) and subjective wellbeing (Tian et al., 2020), few have examined the impact of RS as a multidimensional construct influencing marathon runners’ LS, and little is known about the potential impact of the psychological commitment (PC) and social support (SS) on LS. Regarding the current dilemma, PC has been suggested as a key mediator in the relationship between leisure involvement and flow experience (Cheng et al., 2016). PC reflects individual’s loyalty to the activities in which they involve (Pritchard et al., 1999). In other words, when people are seriously engaged in some leisure activities, they will gain a series of durable benefits which may contribute them to commit to those activities strongly (Stebbins, 2007; Cheng et al., 2016). In addition, SS (e.g., from family or friends) was seen as a key factor that can negotiate individual’s leisure constraints (Brown et al., 2001). Usually, higher degree of SS may contribute to the role of RS on LS.

Therefore, the objectives of this study are triple fold. The first is to access how the behavior, cognition, and affect dimensions of RS influence runners’ LS. The second is to examine the mediating effect of PC on the relationship between dimensions of RS and LS. The third is to test the moderating effect of SS on the relationship between dimensions of RS and LS.

## Literature review and development of hypotheses

### Recreation specialization and life satisfaction

The RS framework originated from Bryan (1977) was firstly used to explain recreational trout fishermen’s attitudinal and behavioral differences. The essence of RS is that outdoor recreation participants usually progress along a continuum from general interest and low engagement to specialized interest and high engagement (Bryan, 2000). Since Bryan’s original research, scholars had made great efforts on how to evaluate RS accurately. A three-dimension specialization model proposed by McIntyre and Pigram (1992) has been widely used, which included dimensions of behavior, cognition, and affect. The behavioral dimension measured prior experience with a specific activity and familiarity with a recreational setting (McIntyre and Pigram, 1992). The cognitive dimension referred to the level of self-assessed knowledge and skill that they have accumulated through significant personal efforts (Waight and Bath, 2014). The affective dimension was characterized by personal commitment and enduring involvement (Buchanan, 1985; McFarlane, 2004).

RS has been successfully applied to examine within-group differences among participants in outdoor leisure sport activities, including marathon (Park et al., 2018), cycling (Lamont and Jenkins, 2013; Shafer and Scott, 2013), camping (McIntyre and Pigram, 1992; McFarlane, 2004), canoe (Wellman and Smith, 1982), and hikers (Kim and Song, 2017). For example, using a latent profile analysis, hikers were divided into three subgroups: novice, affection-driven, and expert; they exhibited significant difference on their satisfaction and revisit intention (Song et al., 2018). In addition, previous studies also reported the role of RS as a dependent variable, an independent variable, or a mediating variable. For example, Cheung et al. (2017) verified that a direct and positive impact was found between RS and pro-environmental attitudes among birdwatchers in Hong Kong.

Although previous studies paid less attention to evaluate the role of RS on LS, they also provided some indirect and reliable evidence. A recent study confirmed a direct influence of RS on cycling participants’ subjective wellbeing, a multidimensional construct consisted of LS, positive and negative experience (Tian et al., 2020). Enduring involvement, seen as affective dimension of RS by McIntyre and Pigram (1992), had a positive direct effect on LS among 10-mile running participants (Sato et al., 2018). Moreover, serious participants reported experiencing higher degree of LS and perceived health than the casual participants (Kim et al., 2011; Heo et al., 2013). In other words, as the level of behavior, cognition, and affect improved continuously in physical active leisure activities, people would be inclined to report a higher satisfaction with life.

### Recreation specialization and psychological commitment

Commitment refers to a process through which an individual becomes dedicated to organize the patterns of their leisure behavior for expressing their needs (Buchanan, 1985). PC was defined as ‘a tendency to be devoted to individual’s activities participation despite alternative options are available’ (Pritchard et al., 1999; Lee and Kyle, 2014). It was seen as an essential element for understanding why individuals choose to participate in a specific leisure activity, or revisit particular places (Backman and Crompton, 1991; Pritchard et al., 1999). Previous studies came to an agreement on dividing PC into four major dimensions: resistance to change, position involvement, volitional choice, and informational complexity (Pritchard et al., 1999; Iwasaki and Havitz, 2004).

As is known to us, individuals may experience complex and sequential psychological processes before becoming loyal participants (Iwasaki and Havitz, 1998).

A previous study indicated that individuals can experience some positive psychological states (e.g., wellbeing and contemplation) from the activities through conquering adversity, and accumulating knowledge, skill, or experience (Stebbins, 2018). In a qualitative research, scholar found that degree of RS acted as a predictor of event attendance, and individuals usually negotiated constraints (e.g., low confidence and no partner) in order to commit to event attendance (Lewis and Moital, 2013). Moreover, Cheng et al. (2016) confirmed that higher enduring involvement (e.g., attraction, self-expression, and centrality to lifestyle) can lead to a higher level of PC by employing a structural equation modeling. Kyle et al. (2004) also indicated that when tourists exhibited higher degree of leisure involvement, they would exhibit strong PC to the activity and place. Based on the statement above, this study deduces that when individuals continuously involved in marathon running, they will accumulate related experiences, identify their preference, and develop a commitment to the activity.

### Psychological commitment and life satisfaction

Previous studies indicated that serious engagement in physically active activities often gained higher level of life satisfaction and health (Kim et al., 2011; Heo et al., 2013). Considering the essence of PC, “continuity” and “resistance to change,” participants with higher degree of PC always acquired many durable benefits as the leisure career processed (Stebbins, 2007). In a longitudinal study, Sato et al. (2015) proposed that achievement and positive experience through running event engagement would be help for evaluating individual’s life satisfaction. In other words, when individuals are strongly psychological commit to marathon events participation, they believe that it can contribute to own a higher LS.

### Mediating role of psychological commitment

Previous studies paid great attention to examine the mediating role of individual’s PC on the relationship between leisure involvement and behavioral loyalty (Iwasaki and Havitz, 2004; Kyle et al., 2004). For example, Kyle et al. (2004) confirmed that dimension of PC had an indirect effect between leisure involvement and behavioral loyalty among hikers along the Appalachian Trail. Bodet (2012) suggested that PC played a mediating role on the relationship between enduring involvement and loyalty in sport participation services. However, they ignored to explore the influence of PC between dimensions of RS and LS.

Existing literature suggested that physically active leisure can improve participants’ quality of life through providing positive experiences, such as psychological involvement and flow experience (Sato et al., 2014; Cheng et al., 2016). As discussed in previous paragraphs, dimensions of RS (i.e., behavior, cognition, and affect) may have a positive impact on PC and LS (Kyle et al., 2004; Heo et al., 2013; Cheng et al., 2016; Sato et al., 2018); PC may positively associate with LS (Sato et al., 2015). Based on the statement above, this study suggested that runners will commit to engage in marathon event tourism as degree of their RS increases. They will conquer the difficulties and constraints, develop a higher PC, and gain more positive experience and durable outcome related to LS.

### Social support as a moderator

SS generally refers to the ‘process of interaction in relationship which improve, coping, esteem, belong, and competence through actual or perceived exchanges of physical of psychosocial resources’ (Gottlieb, 2000). SS can contribute to individual’s health outcome by modifying the effects of a stressful situation or impacting on health directly (House et al., 1988; Thoits, 1995). According to this opinion, SS can encourage individuals to maintain and initiate running activities by psychological variables such as self-efficacy. It also suggested that SS can provide important information or material resources which can increase degrees of participation. In addition, previous studies also suggested dimensions of RS may influence the evaluation of individual’s LS (Kim et al., 2011; Heo et al., 2013). Considering these arguments above, the current study proposes that SS can strength the relationship between behavior, cognition, affect, and LS.

**Figure 1:**
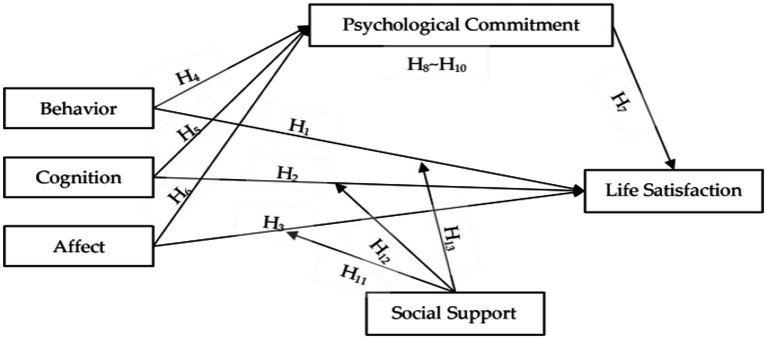
Proposed conceptual model.

In this study, we aimed at examining the relationship between dimensions of RS, PC, LS, and SS among Chinese marathon tourists. Based on the ideas mentioned above, we proposed a conceptual hypotheses model that is shown in Figure 1.

*H*_*1*_: Marathon runners’ behavior of RS has a positive influence on their LS.

*H*_*2*_: Marathon runners’ cognition of RS has a positive influence on their LS.

*H*_*3*_: Marathon runners’ affect of RS has a positive influence on their LS.

*H*_*4*_: Marathon runners’ behavior of RS has a positive influence on their PC.

*H*_*5*_: Marathon runners’ cognition of RS has a positive influence on their PC.

*H*_*6*_: Marathon runners’ affect of RS has a positive influence on their PC.

*H*_*7*_: Marathon runners’ PC has a positive influence on their LS.

*H*_*8*_: Marathon runners’ PC plays a mediating role between their behavior of RS and their LS.

*H*_*9*_: Marathon runners’ PC plays a mediating role between their cognition of RS and their LS.

*H*_*10*_: Marathon runners’ PC plays a mediating role between their affect of RS and their LS.

*H*_*11*_: Marathon runners’ SS plays a moderating role between their behavior of RS and their LS.

*H*_*12*_: Marathon runners’ SS plays a moderating role between their cognition of RS and their LS.

*H*_*13*_: Marathon runners’ SS plays a moderating role between their affect of RS and their LS.

## Methodology

### Measurements

RS was measured with 9 items modified from recent studies (Song et al., 2018; Tian et al., 2020); It was used to evaluate the degree of specialization for marathon running participants. The scale included three dimensions: behavior (3 items), cognition (2 items), and affect (4 items). The statement for affect was as follows: “If stopped running, I would lose touch with my friends.” Both the behavioral and cognitive dimensions were rated on a 5-point Likert scale where “1” represents “novice” and “5” represents “expert.” The affect dimension was assessed with a 5-point Likert scale where “1” represents “disagree strongly” and “5” represents “agree strongly.”

Satisfaction With Life Scale (SWLS) which developed by Diener et al. (1985) was applied to measure the degree which a person positively evaluates the overall quality of his/her life. The Chinese-version SWLS was confirmed with good reliability and validity by Xiong and Xu (2009). The scale consists of five items with 7-point Likert scale where “1” represents “disagree strongly” and “7” represents “agree strongly.” The statement for LS was as follows: “In most ways my life is close to my ideal.”

Psychological Commitment Scale (PCS), modified from Pritchard et al. (1999) and Kyle et al. (2004), was used to measure the extent of commitment for marathon runners. A recent study reported that this scale appeared to exhibit satisfactory measurement qualities (Cheng et al., 2016). It included 10 items, covering four dimensions: resistance to change (3 items), position involvement (3 items), volitional choice (2 items), and information complexity (2 items). The statement for resistance to change was as follows: “My preference to participate in running will not willingly change.” A 7-point Likert scale was used where “1” represents “disagree strongly” and “7” represents “agree strongly.”

The Multidimensional Scale of Perceived Social Support (MSPSS), developed by Zimet et al. (1988), was applied to measure the respondent’s social support. The MSPSS consisted of 12 items, reducing to three dimensions: significant other (4 items), friends (4 items), and family (4 items). The statement for significant other was as follows: “There is a special person in my life who cares about my feelings.” The MSPSS was rated with a 7-point Likert scale where 1 represents “disagree strongly” and 7 represents “agree strongly.”

According to the experiences of existing literature (Qiu et al., 2020; Tian et al., 2022), five demographic variables and a behavioral variable were introduced as covariate variables in the proposed model. They are gender, age, marital status, income, education, and the number of running event participation per year.

### Procedures and date analysis

The data for this study were collected at the 2021 Xiamen International Marathon Event which was held on April 10 in Xiamen (a tourism city in southeast China). This event was recently certified as a world athletics elite platinum label event by International Athletics Federation, and has successfully attracted over 15,000 participants every year since 2015. Based on the size of the event, this study distributes the questionnaire randomly (i.e., every 20th person) to marathon runners near the finish area. The respondents were suggested to sign the informed consent before they start to fill out the questionnaire. A total of 393 questionnaires were collected through sharing a QR code from a web-based platform (i.e., Questionnaire Star), but 34 of the sample were excluded from the data analysis according to Gibson’s (1998) definition of sport tourism, because they were not temporarily outside of their home communities. Finally, 359 questionnaires were used to examine all the research hypotheses.

SPSS 23.0 and JASP 0.16 were performed to analyze the data in this study. Parametric analyses, seen as the standard tools of psychological statistics (Norman & Anderson, 1961), were “robust” as judged from the observation that parametric and non-parametric analyses lead to similar results regarding statistical significance (Mircioiu and Atkinson, 2017). So parametric procedure was used to examine related data in this study. Specifically, the respondent profile, mean, and standard deviations were evaluated by descriptive analysis. Reliability of all the variables in this study was accessed using Cronbach’s alpha (CA). The correlation of all the variables was evaluated by Pearson’s correlation coefficient. Confirmatory Factor Analysis in JASP was performed to test the convergent validity and discriminant validity of all the subscales. Regression analysis was applied to examine hypothesis 1 to hypothesis 7. Process V 4.0 developed by Hayes et al. (2017) was used to decide hypothesis 8 to hypothesis 13.

## Results

### Respondent profile

Table 1 reports the profiles of the respondents. As a whole, most respondents were middle age, married, well-educated, and higher income.

**Table 1:** Respondent profile.

Variable	Characteristics	Frequency (n)	Percentage (%)
Gender	Male	251	69.9
	Female	108	30.1
Age	18–29	109	30.4
	30–44	115	32.0
	45–60	93	25.9
	61 and above	42	11.7
Marital status	Unmarried	82	22.8
	Married	251	69.9
	Divorced or widowed	26	7.2
Education	High school or below	21	5.8
	College or university	257	71.6
	Postgraduate	81	22.6
Income (/year)	US$3,000 and below	33	9.2
	US$3,001–US$7,500	64	17.8
	US$7,501–US$18,000	183	51.0
	US$18,000 and above	79	22.0
Event participant (/year)	1 to 2 times	65	18.1
	3 to 4 times	160	44.6
	5 to 6 times	107	29.8
	6 times above	27	7.5
Total		359	100

### Descriptive statistics

Before testing the research hypotheses, the reliability and validity of all constructs were examined by using JASP 0.16 in this study. As reported in Table 2, the internal consistency values ranged from 0.72 to 0.95, indicative of reliable consistency among the items in each subscale (Nunnally and Bernstein, 1994). Convergent validity reflects the extent to which a set of items possess the properties expected of the focal construct (Nunnally, 1967; Bagozzi and Yi, 2012). Three indicators, including factor loading (FL), composite reliability (CR), and average variance extracted (AVE), were used to evaluate the convergent validity of each construct. As shown in Table 2, the FLs for most items were high than 0.70 (i.e., 0.74 to 0.93), except for one item in the subscale of SS (FRI2 = 0.34). According to the suggestion of Hair et al. (2011), when an item’s FL is below 0.70, it should be removed from the scale for increasing the CR. In this study, the reported CR values ranged from 0.71 to 0.92, and the AVE values ranged from 0.55 to 0.90. These indicators met the acceptable threshold of an AVE value higher than 0.50 (Barclay et al., 1995) and a CR value higher than 0.70 (Fornell and Larcker, 1981).

**Table 2:** FL, CA, AVE, and CR of each construct.

Constructs	Code	Items	FL	CA	AVE	CR
Specialization						
Behavior	BEH1	How long do you use for each run?	0.74	0.72	0.60	0.82
	BEH2	How many times do you run every week?	0.79			
	BEH3	How many years have you been running?	0.79			
Cognition	COG1	Knowledge of running	0.93	0.85	0.90	0.92
	COG2	Running skill	0.91			
Affection	AFF1	Running is very important to me	0.74	0.86	0.61	0.86
	AFF2	I find that much of my life is organized around running	0.83			
	AFF3	If stopped running, I would lose touch with my friends	0.80			
	AFF4	I would rather go running than do other activities	0.76			
Psychological commitment						
Resistance to change	REC1	My preference to participate in running will not willingly change	0.75	0.88	0.71	0.88
	REC2	It would be difficult to change my beliefs about running	0.87			
	REC3	Even if close friends recommended another pastime, I would not change my preference for running	0.90			
Position involvement	POI1	I prefer to participate in running because their image of the activity comes closest to reflecting my lifestyle	0.81	0.80	0.59	0.81
	POI2	When I participate in running it reflects the kind of person I am	0.78			
	POI3	I prefer to participate in running because provider’s service makes me feel important	0.71			
Volitional choice	VOC1	My decision to participate in running was freely chosen from several alternatives	0.70	0.75	0.55	0.71
	VOC2	I am fully responsible for the decision to participate in running	0.78			
Information complexity	INC1	I consider myself to be an educated consumer regarding running	0.86	0.86	0.75	0.86
	INC2	I am knowledgeable about running	0.87			
Life Satisfaction	LS1	In most ways, my life is close to my ideal	0.85	0.90	0.67	0.91
	LS2	The conditions of my life are excellent	0.80			
	LS3	I am satisfied with my life	0.85			
	LS4	So far, I have gotten the important things I want in life	0.85			
	LS5	If I could live my life over, I would change almost nothing	0.72			
Social support				0.89	0.67	0.89
Significant other	SIO1	There is a special person who is around when I am in need	0.86			
	SIO2	There is a special person with whom I can share joys and sorrows	0.76			
	SIO3	I have a special person who is a real source of comfort to me	0.81			
	SIO4	There is a special person in my life who cares about my feelings	0.83			
Friends	FRI1	My friends really try to help me	0.75	0.84^b^	0.64^b^	0.84^b^
	FRI2^a^	I can count on my friends when things go wrong	0.34			
	FRI3	I have friends with whom I can share my joys and sorrows	0.86			
	FRI4	I can talk about my problems with my friends	0.79			
Family	FAM1	My family really tries to help me	0.81	0.84	0.65	0.88
	FAM2	I get the emotional help & support I need from my family	0.77			
	FAM3	I can talk about my problems with my family	0.86			
	FAM4	My family is willing to help me make decisions	0.79			

FL = factor loading; CA = Cronbach’s alpha; AVE = average variance extracted; CR = composite reliability; All the factor loadings are significant at the 0.001 level of significance.

a Items have been excluded because the factor loadings were below 0.7.

b The values were computed after exclusion of the deleted items.

Table 3 presents the results of descriptive statistics and discriminant validity for each subscale. Affect had a higher mean score (*M* = 3.76, *SD* = 0.84) on the scale of RS, followed by cognition (*M* = 3.60, *SD* = 0.91); behavior had a lower mean score (*M* = 2.72, *SD* = 0.96). These results were consistent with the findings of a recent research (Tian et al., 2020). In terms of the PC scale, a higher mean score was found for volitional choice (*M* = 5.62, *SD* = 1.04), followed by resistance to change (*M* = 5.53, *SD* = 1.07) and position involvement (*M* = 5.43, *SD* = 1.04); information complexity had a lower mean score (*M* = 5.33, *SD* = 1.14). These results indicated that the respondents exhibited higher attitudinal loyalty for marathon running. On the scale of LS, the respondents also reported higher life satisfaction (*M* = 5.25, *SD* = 1.08), which indicated that the respondents were satisfied with the quality of their current life. As for the scale of SS, family had a higher mean score (*M* = 5.63, *SD* = 0.99), followed by friends (*M* = 5.52, *SD* = 1.07); significant other had a lower mean score (*M* = 5.45, *SD* = 1.16).

**Table 3:** Descriptive statistics and discriminant validity.

Constructs	M	SD	BEH	COG	AFF	REC	POI	VOC	INC	SWL	SIO	FRI	FAM
BEH ^a^	2.72	0.96	**0.77**	0.29	0.35	0.34	0.26	0.17	0.35	0.15	0.07	0.08	0.08
COG^a^	3.60	0.91		**0.91**	0.60	0.41	0.43	0.35	0.59	0.44	0.29	0.32	0.37
AFF^b^	3.76	0.84			**0.93**	0.55	0.57	0.40	0.55	0.41	0.33	0.36	0.38
REC^c^	5.53	1.07				**0.94**	0.83	0.69	0.75	0.56	0.47	0.50	0.56
POI^c^	5.43	1.04					**0.89**	0.73	0.78	0.61	0.55	0.58	0.59
VOC^c^	5.62	1.04						**0.75**	0.59	0.57	0.51	0.55	0.58
INC^c^	5.33	1.14							**0.93**	0.62	0.47	0.48	0.52
LS^b^	5.25	1.08								**0.95**	0.59	0.60	0.61
SIO^c^	5.45	1.16									**0.94**	0.82	0.70
FRI^c^	5.52	1.07										**0.86**	0.77
FAM^c^	5.63	0.99											**0.92**

The correlation coefficients are shown in the upper diagonals. The square roots of the AVE values are shown in boldface type in the diagonal.

a Rated on a 5-point scale from 1 (novice) to 5 (expert).

b Rated on a 5-point scale from 1 (disagree strongly) to 5 (agree strongly).

c Rated on a 7-point scale from 1 (disagree strongly) to 7 (agree strongly).

Discriminant validity reflects the degree to which a group of variables meant to measure a construct can differentiate the construct from others in the model. It can be examined by comparing the square root of the AVE and correlation between the constructs (Fornell and Larcker, 1981). As shown in Table 3, the square roots of the AVE of all the subscales were high than the correlation coefficient of other constructs (See the diagonal versus non-diagonal elements), which suggested all the constructs showed acceptable discriminant validity.

In addition, all constructs used in this study had significantly positive correlations with each other ranging from 0.07 to 0.83, *p* < 0.01.

### Hypothesis testing results

The multiple regression was performed to examine the hypothesis 1 to hypothesis 7. The statistical results confirmed that behavior (*β* = 0.15, *p <* 0.01), cognition (*β* = 0.35, *p* < 0.001), affect (*β* = 0.28, *p* < 0.001), and PC (*β* = 0.59, *p* < 0.001) have positive and significant effects on LS. Thus, hypotheses H1, H2, H3, and H7 are supported. Similarly, the results confirmed that behavior (*β* = 0.22, *p* < 0.001), cognition (*β* = 0.38, *p* < 0.001), and affect (*β* = 0.43, *p* < 0.001) have positive and significant impacts on PC. These findings support hypotheses H_4_ to H_6_.

### The mediating role of psychological commitment

As Table 4 illustrates, the indirect effects were estimated and the 95% confidence intervals were calculated. Specifically, the mediating role of PC on the relationship between behavior, cognition, affect, and LS was significant as zero was not included in any of the confidence intervals. That is (a*b = 0.09, LB = 0.05, UB = 0.14), (a*b = 0.14, LB = 0.08, UB = 0.20), and (a*b = 0.20, LB = 0.11, UB = 0.29), respectively. Following the suggestion of Wen and Ye (2014), it was confirmed that PC fully mediated the relationship between behavior, cognition, and LS. However, PC partially mediated only the relationship between affect and LS. Therefore, these findings indicate that PC has a mediating effect between behavior, cognition, affect, and LS, confirming hypotheses H_8_ to H_10_.

**Table 4:** Mediating effect of PC.

No.	Hypothesis	IPC	BootSE	95% CI		DPC	Decision
				Lower	Upper		
H_8_	Behavior → PC → SWL	0.09	0.03	0.05	0.14	0.08	FMS
H_9_	Cognition → PC → SWL	0.14	0.03	0.08	0.20	0.21	FMS
H_10_	Affect → PC → SWL	0.20	0.04	0.11	0.29	0.08^*^	PMS

IPC = indirect path coefficient; DPC = direct path coefficient; FMS = full mediation supported; PMS = partial mediation supported.

### The moderate role of social support

The moderation role of SS on the relationship between dimensions of RS and LS was tested as shown in Table 5. For analysis, three interaction variables were produced, including SS × behavior, SS × cognition, and SS × affect. According to the results, the effect of SS × affect on LS was significant (*β* = 0.10, *p* < 0.05). However, the effect of SS × behavior (*β* = 0.03, *p >* 0.05) and SS × cognition (*β* = 0.06, *p* > 0.05) on LS was not significant. In other words, SS can significantly promote the relationship between affect and LS. While SS did not provide any significant impact for the role of behavior and cognition on LS.

**Table 5:** Moderating effect of SS.

No.	Hypothesis	*β*	*p*	95% CI		Decision
				Lower	Upper	
H_11_	SS × Behavior → LS	0.03	0.48	−0.05	0.11	Not supported
H_12_	SS × Cognition→ LS	0.06	0.16	−0.02	0.14	Not supported
H_13_	SS× Affect → LS	0.10	0.03	0.01	0.20	Supported

## Discussion

The present study extends existing literature on runner’s RS toward marathon event tourism in three main ways. First, we sought to clarify how RS dimension (i.e., behavior, cognition, and affect) and runners’ PC are related. Second, this study examined the mediating effect of PC on the relationship between RS dimensions and LS. Third, the current study investigated the moderating role of SS between RS dimensions and LS. A proposed conceptual model was designed to examine the relationship between dimensions of RS, PC, LS, and SS among marathon event tourists in China. The standardized estimate results for this study supported 11 out of the 13 hypotheses, with H_11_ and H_12_ as the exception.

As shown in Table 6, this study fills the gap in the existing literature by revealing a significant and positive influence of runners’ RS dimensions on their LS. The findings were consistent with previous contributions that have shown a link between behavior, cognition, affect dimension, and runners’ LS (Heo et al., 2013; Sato et al., 2018). Activity theory claimed that the amount or frequency of activity participation and the level of attachment related to an activity could impact a person’s LS (Lemon et al., 1972). This research also extended to understand the antecedent factors of LS. As mentioned in previous studies, when individuals exhibited a serious level of behavioral engagement, knowledge acquirement, and emotional attachment, their LS always increased correspondingly (Kim et al., 2011; Heo et al., 2013).

**Table 6:** Main regression results in the proposed research model.

No.	Hypothesis	*β*	*t*-value	Decision
H_1_	Behavior → LS	0.15^**^	2.93	Supported
H_2_	Cognition → LS	0.35^***^	7.66	Supported
H_3_	Affect → LS	0.28^***^	5.68	Supported
H_4_	Behavior → PC	0.22^***^	4.90	Supported
H_5_	Cognition → PC	0.38^***^	8.84	Supported
H_6_	Affect → PC	0.43^***^	10.09	Supported
H_7_	PC → LS	0.59^***^	12.64	Supported

** *p* < 0.01;

*** *p* < 0.001.

According to study results, RS dimensions were significantly and positively related to runners’ PC. The results enrich existing research of Kyle et al. (2004), Cheng et al. (2016), and Ridinger et al. (2012), which confirmed that tourists showed strong PC to their pursuits when they presented a high level of leisure involvement. Recent suggested that individual’s self-discipline behavior and curiosity on activity can make them acquire a series of physical, psychological, and social benefits, which may indirectly strengthen their degree of PC (Stebbins, 2018). Moreover, the findings of this study provided some supports to Sato et al. (2015) and Stebbins (2007) finding that positive experience was positively associated with people’s LS.

Study results revealed that runners’ PC can play a mediating effect between RS dimensions and LS. Previous research have confirmed the mediating role of PC between leisure involvement and flow experience, and consumer loyalty (Iwasaki and Havitz, 2004; Bodet, 2012; Cheng et al., 2016). By developing a structural modeling, Lee and Scott (2006) suggested that as individual became increasing specialized, the benefits they obtained far outweighed any costs they might experience along the way. In addition, high quality of life is always rooted in a capacity to search deep satisfaction and fulfillment through experiencing with serious leisure to eventually carve out an optimal leisure lifestyle (Stebbins, 2007). Therefore, the findings in this study contribute to understand the connection between RS dimensions and LS, especially the indirect effect of PC on the relationship between behavior, cognition and LS.

Extending to previous study (Ricciardo et al., 2006), this study demonstrated that affect dimension interacted with SS when it predicted individual’s LS. Existing literature confirmed that positive social influences had a positive influence on individual’s negotiation efforts among the process they become specialized (Kim et al., 2019), they played a key role in achieving diverse durable benefits and developing unique leisure identities (Lyu and Oh, 2015). Therefore, higher social support can strengthen the positive role of affect dimension on runner’s LS. However, SS did not significantly moderate the role of behavior and cognition on runners’ LS. In other words, individuals may use other negotiation strategies related to intrapersonal and structural constraints for pursuing a satisfied life when they processed toward a higher level of specialization (Park et al., 2017).

Although this study provides valuable insight into the relationship between dimension of RS, PC, LS, and SS in a leisure background, there are some limitations as follows. First, the findings in this study were confirmed under the background of COVID-19 pandemic, which has impacted all aspects of individual’s everyday life, especially the experience of event participant and evaluation on their life. Hence, the results should be re-examined after conquering the COVID-19 pandemic. Second, while the proposed conceptual model has been carefully designed, this study may still ignore variables such as self-efficacy, leisure motivation, and leisure constraints. It should explore the influence of these variables on the theoretical framework in the future. Third, this study collected data from a random convenience sample of marathon runners in Xiamen Marathon Event. Future study should recruit the respondents from a wider geographic area and other types of activities.

## Conclusion

Physically active leisure plays an important role in promoting individual’s LS (Sato et al., 2015, 2018). Study results contributed to existing literature by confirming the positive influence of behavior, cognition, affect, PC on runners’ LS, and the moderating role of SS with affect on runners’ LS. Considering the normalization of the COVID-19 pandemic, these results could open a new door to the leisure and recreation manager, marathon event organizer, and marathon participants for weaving a satisfied everyday life.

From a theoretical perspective, the findings support the positive impact of physically active leisure participation on experiencing higher level of LS. These results also extended the existing literature (Kim et al., 2011; Sato et al., 2018) by examining the mediating effect of PC, and the moderating role of SS. In addition, they also contribute to understand the predictors and influential paths of LS. From a practical perspective, individuals can experience a high degree of LS from being specially engaged in physically active leisure. In terms of individuals, they should make significant personal effort to conquer adversity, accumulate knowledge and skill, and form a long-lasting leisure career. For providers of leisure sport service, improving the quality of events, popularizing the sport skill and knowledge, and encouraging social group culture may be the best way for improving individual’s LS.

## Data availability statement

The raw data supporting the conclusions of this article will be made available by the authors, without undue reservation.

## Ethics statement

Ethical review and approval was not required for the study on human participants in accordance with the local legislation and institutional requirements. Written informed consent from the patients/ participants or patients/participants legal guardian/next of kin was not required to participate in this study in accordance with the national legislation and the institutional requirements.

## Author contributions

YQ: methodology, project administration, and funding acquisition. HT: formal analysis, writing—original draft preparation, and writing—review and editing. WZ: investigation. HT and WZ: data curation. All authors contributed to the article and approved the submitted version.

## Funding

This research was supported by the Starting Research fund from Shaoxing University (No. 20216011), the Hengyi foundation of Zhejiang University, and national social science funding in China (No. 16BTY077).

## Conflict of interest

The authors declare that the research was conducted in the absence of any commercial or financial relationships that could be construed as a potential conflict of interest.

## Publisher’s note

All claims expressed in this article are solely those of the authors and do not necessarily represent those of their affiliated organizations, or those of the publisher, the editors and the reviewers. Any product that may be evaluated in this article, or claim that may be made by its manufacturer, is not guaranteed or endorsed by the publisher.
